# The Melanoma care study: protocol of a randomised controlled trial of a psycho-educational intervention for melanoma survivors at high risk of developing new primary disease

**DOI:** 10.1186/s40359-015-0074-3

**Published:** 2015-07-11

**Authors:** Mbathio Dieng, Nadine A. Kasparian, Rachael L. Morton, Graham J. Mann, Phyllis Butow, Scott Menzies, Daniel S.J. Costa, Anne E. Cust

**Affiliations:** Cancer Epidemiology and Services Research, Sydney School of Public Health, The University of Sydney, The Lifehouse, Level 6 North, 119-143 Missenden Road, Camperdown, NSW 2050 Australia; Discipline of Paediatrics, School of Women’s and Children’s Health, UNSW Medicine, The University of New South Wales, Sydney, NSW 2031 Australia; Sydney School of Public Health, The University of Sydney, Sydney, NSW 2006 Australia; Westmead Institute for Cancer Research, University of Sydney at Westmead Millennium Institute and Melanoma Institute Australia, Sydney, NSW 2145 Australia; Centre for Medical Psychology and Evidence-based Decision-making, School of Psychology, University of Sydney, Sydney, NSW 2006 Australia; Discipline of Dermatology, The University of Sydney at The Sydney Melanoma Diagnostic Centre, Royal Prince Alfred Hospital, Camperdown, NSW 2050 Australia; Psycho-oncology Co-operative Research Group, School of Psychology, University of Sydney, Sydney, NSW 2006 Australia

**Keywords:** Melanoma, Psycho-education, Cancer, Recurrence, Protocol, Randomised controlled trial, Psychological stress, Intervention

## Abstract

**Background:**

Despite a good prognosis for most melanoma survivors, many experience substantial fear of new or recurrent melanoma, worry and anxiety about the future, and unmet healthcare needs. In this protocol, we outline the design and methods of the Melanoma Care Study for melanoma survivors at high risk of developing new primary disease. The objective of this study is to evaluate the efficacy and cost-effectiveness of a psycho-educational intervention for improving psychological and behavioural adjustment to melanoma risk.

**Design:**

The study design is a two-arm randomised controlled trial comparing a psycho-educational intervention to usual care.

**Methods:**

The intervention is comprised of a newly-developed psycho-educational booklet and three telephone sessions delivered by a trained psychologist. A total of 154 melanoma survivors at high risk of developing new primary disease who are attending one of three melanoma high risk clinics in New South Wales, Australia, will be recruited. Participants will be assessed at baseline (6 weeks before their high risk clinic dermatological appointment), and then 4 weeks and 6 months after their appointment. If effectiveness of the intervention is demonstrated at 6 months, an additional assessment at 12 months is planned. The primary outcome is fear of new or recurrent melanoma, as assessed by the Fear of Cancer Recurrence Inventory (FCRI). Secondary outcomes include anxiety, depression, unmet supportive care needs, satisfaction with clinical care, knowledge, behavioural adjustment to melanoma risk, quality of life, and cost-effectiveness of the intervention from a health system perspective. Following the intention-to-treat principle, linear mixed models will be used to analyse the data to account for repeated measures. A process evaluation will also be carried out to inform and facilitate potential translation and implementation into clinical practice.

**Discussion:**

This study will provide high quality evidence on the efficacy and cost-effectiveness of a psycho-educational intervention aimed at improving psychological and behavioural adjustment amongst melanoma survivors at high risk of new primary disease.

**Trial registration:**

ACTRN 12613000304730

## Background

Despite a strong awareness of skin cancer prevention and early detection, Australia still has the highest incidence of melanoma in the world. With more than 12,500 cases diagnosed annually in Australia, melanoma is the fourth most common cancer (Australian Cancer Incidence and Mortality (ACIM) 2010). Melanoma detected at an early stage has a 5-year survival rate of greater than 90 %; however, this decreases to around 50 % with diagnosis at a late stage (Balch et al. 2009). In Australia, the burden of melanoma is considerable. It is the eighth most common cause of death from cancer, causing more than 1500 deaths per year and accounting for 22,800 disability-adjusted life years (DALYs). Of these, 17,200 are years lost due to premature death and 5600 are years of healthy life lost due to disease, disability or injury (Australian Cancer Incidence and Mortality (ACIM) 2010; Australian Institute of Health and Welfare & Australasian Association of Cancer Registries 2012).

Once a melanoma has been detected, the risk of developing another melanoma is much higher. A person who has had one previous melanoma has a 9-fold risk of developing a new primary melanoma compared to the average person in Australia, and the risk remains elevated more than 20 years after the initial melanoma diagnosis (Bradford et al. 2010). Risk of recurrence (i.e. that the melanoma will spread to another part of the body) is related to the clinical features of a person’s melanoma, and is estimated overall to be 9 % (Bradford et al. 2010).

A melanoma diagnosis is often perceived as life-threatening and can trigger psychological distress, particularly amongst those at higher risk of recurrence. Research involving melanoma survivors has found that fear of new or recurrent cancer (FCR) (Vickberg 2003) and anxiety are common among this group (Kasparian et al. 2009; McLoone et al. 2013a). FCR is also common among other cancer survivors, with a review showing 42–70 % report levels of FCR warranting clinical assessment (Simard & Savard 2009; Thewes et al. 2012).

There is evidence of a significant negative correlation between FCR and psychological well-being and health-related quality of life (Koch et al. 2013; Sarkar et al. 2014). Even years after the initial diagnosis, FCR may reduce health-related quality of life and induce psychosocial morbidity (Koch et al. 2013); however, prospective studies are lacking. Further research is required to more fully understand the relationship between FCR and other factors, such as anxiety, distress, quality of life, coping, as well as unmet needs, satisfaction with clinical care, and health behaviours.

Despite a large proportion of high risk melanoma survivors reporting persistent fear and uncertainty about the possibility of new disease, disease recurrence or metastases, few appear to receive professional psychological support for melanoma-related concerns (McLoone et al. 2012). Qualitative research (Lee-Jones et al. 1997) suggests that people with melanoma tend to feel they do not belong with other cancer patients in support groups, and that their fears and concerns may not be understood by family and friends because of their seemingly healthy outward appearance and ‘straight forward’ treatment.

Therefore, management of FCR requires educational and psychotherapeutic approaches that address emotional, social, behavioural and cognitive responses to cancer. Research has shown that allowing cancer patients to discuss their fears may help reduce threats to their emotional well-being (Lee-Jones et al. 1997). Several interventions have been developed in the past to facilitate behaviour change, enhance participants’ coping and adjustment to melanoma, and improve patient satisfaction with clinical care (McLoone et al. 2013a). However, no studies have developed and evaluated psychological support interventions specifically designed for melanoma survivors at high risk of new primary disease. To address this gap in psychological support, our team developed a supportive care program designed for melanoma survivors at high risk of developing new primary disease. The intervention is multi-faceted and is broadly designed to meet supportive needs. This study will evaluate both the efficacy and cost-effectiveness of the newly developed psycho-educational intervention.

## Methods

### Study aims and hypotheses

The objective of the study is to improve emotional, social, behavioural and cognitive adjustment to melanoma risk amongst melanoma survivors at high risk of developing new primary disease. The primary aim of the study is to evaluate, using a randomised controlled trial, the efficacy of a psycho-educational intervention in reducing fear of new or recurrent melanoma, as measured by the Fear of Cancer Recurrence Inventory (FCRI), compared to usual care. The secondary aims are to:

- Evaluate the effect of the intervention on anxiety, depression, unmet supportive care needs, satisfaction with clinical care, knowledge, quality of life, and behavioural adjustment to melanoma risk (sun exposure, sun protection, and skin self-examination);
- Evaluate the cost-effectiveness of the intervention within the Australian health system; and
- Conduct a process evaluation to try to identify the ‘active ingredients’ of the newly developed intervention, to inform and facilitate potential translation and implementation into clinical practice.

We hypothesise that compared with those who receive usual care (controls), those who receive the newly developed, tailored psycho-educational intervention will:

- Report a lower severity sub-scale score on the Fear of Cancer Recurrence Inventory (Simard & Savard 2009);
- Have greater understanding of melanoma risk, greater satisfaction with clinical care, lower depression, anxiety and stress, healthier behavioural adjustment to melanoma risk, fewer unmet supportive care needs, and better health-related quality of life.

Additionally we hypothesise that the intervention will be cost-effective from a health system perspective.

## Design

The Melanoma Care Study is a two-arm randomised controlled trial in which an efficacy evaluation, an economic evaluation, and a process evaluation will be carried out. The Template for Intervention Description and Replication (TIDieR) checklist and guide, (Hoffmann et al. 2014) which builds on the CONSORT/SPIRIT statements, (Campbell et al. 2004) will be used to report the efficacy and cost-effectiveness of the intervention. The trial is registered with the Australian and New Zealand Clinical Trials Registry (Registration Number: ACTRN12613000304730). Ethics approval has been obtained from all relevant Ethics Committees, including the Sydney Local Health District (RPAH zone) Ethics Review Committee, the Department of Health and Ageing Human Research Ethics Committee, the University of Sydney ethics committee and the Australian Institute of Health and Welfare Ethics Committee.

### Participants

Participants will be eligible for the study if they meet all of the following criteria:

- Previously diagnosed with melanoma stage 0, I or II;
- At high risk of developing new primary melanoma (attending a high risk clinic);
- Able to give informed consent for the study;
- Possess sufficient English language skills to read the booklet and complete the study questionnaires without an aide; and
- Aged 18 years or older.

Individuals will not be eligible to take part if they meet any one of the following criteria:

- Current stage III or IV (metastatic) melanoma, as research suggests that these patients may have different psychosocial needs to stage 0/I/II patients (where the melanoma has been confined to a primary tumour only);
- At high risk of melanoma but have never had the disease (e.g. people without melanoma who carry a high penetrance genetic mutation);
- Have a known past or current diagnosis of severe major depression, active psychotic illness, or other serious psychiatric condition or cognitive deficit (e.g. dementia).

There is no intention to ‘screen’ individuals for FCR prior to study enrolment. This decision is based on previous evidence accumulated by our team over the past 10 years, showing that people at high risk of melanoma report a range of difficulties across various domains in addition to FCR, including but not limited to, unmet health information needs, practical issues regarding their melanoma care, difficulties communicating with their healthcare team, and challenges in accessing timely and appropriate psychological support. These difficulties are considered important to address in the context of a psycho-educational intervention, irrespective of self-reported FCR scores and thus, screening is regarded as unnecessary in this trial. In addition, the ability of the 9-item FCRI severity subscale to screen for clinical need, and the appropriate cut-points to use, are yet to be demonstrated.

### Recruitment procedures

Individuals who meet the eligibility criteria will be recruited from melanoma high risk clinics (HRC) across New South Wales, Australia. These clinics provide a specialised clinical management service for people at very high risk of developing further primary melanoma (Moloney et al. 2014). This includes patients ≥18 years of age who belong to one or more of the following groups:

- Personal history of ≥1 invasive melanoma and dysplastic nevus syndrome; or
- Personal history of ≥2 primary invasive melanomas, with at least one occurring in the 10 years prior to study recruitment; or
- Personal history of ≥1 invasive melanoma and a family history of at least three first- or second-degree relatives with a confirmed history of melanoma; or
- Confirmed carrier of a *CDKN2A* or *CDK4* gene mutation (the highest penetrance susceptibility gene mutations for melanoma). No history of invasive melanoma is required for this group.

The first HRC was established in 2006 at the Sydney Melanoma Diagnostic Centre at the Royal Prince Alfred Hospital in metropolitan Sydney. In 2012, the service expanded to include clinics at the Melanoma Institute Australia in metropolitan Sydney and the Newcastle Skin Check Clinic in a regional, coastal area; all three of these clinics were sources of recruitment for this study. In late 2014, another HRC opened at Westmead Hospital in metropolitan Sydney. Participants attend these clinics at least 6-monthly for skin monitoring via a variety of clinical imaging techniques, including dermoscopy, digital dermoscopy and total body photography. Individuals are managed according to the assessment of any lesions detected (Moloney et al. 2014). Education in skin self-examination techniques is also provided where appropriate and a record of skin self-examination is maintained by the individual.

**Fig. 1:**
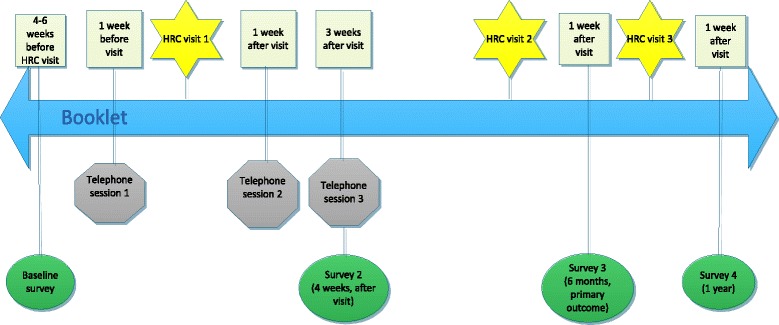
Schedule for study questionnaires and telephone-based sessions with a psychologist

The study invitation package comprising invitation letter, participant information sheet, consent forms, participation card, and reply paid envelope will be sent as a bulk mail out to all eligible HRC patients. The letter of invitation will be signed by each patient’s treating clinician. Participants will be able to opt into the study by returning the signed consent form and participation card, or to decline study participation by returning the non-participation card. Participants who do not return their consent form or participation card within two weeks will receive reminder telephone calls. If the participant does not return their consent form after these telephone calls, no further contact will be made. Participants who opt-in by returning their signed consent form will be contacted by the study coordinator who will provide details and timing of the study materials they will receive, which will be timed according to their next HRC appointment date (see Fig. 1).

### Randomisation

Randomisation will be performed ensuring allocation concealment using the telephone randomisation service at the National Health and Medical Research Council of Australia (NHMRC) Clinical Trials Centre, The University of Sydney. Recruited participants will be assigned an ID code and randomised using minimisation, stratified by HRC site.

To minimise potential contamination, no components of the intervention will be made available to patients other than those in the intervention group until after all data have been collected and the trial is completed. Some of the HRC clinicians have been involved in developing and reviewing intervention components, but they will not be provided with a personal copy and will be asked not to initiate conversation specifically about the intervention with their patients. For ethical reasons, we cannot preclude clinician communication with ‘control’ participants about topics covered by the intervention. Indeed this may happen regardless of the trial due to education from other sources; however, time constraints in the clinic setting are highly likely to minimise contamination among controls.

### The intervention: melanoma care program

The intervention is comprised of two components: a newly-developed psycho-educational booklet, and three individual, telephone-based sessions facilitated by a psychologist.

The *psycho-educational booklet* entitled, ‘Melanoma: Questions and Answers’ is a 76-page, full-colour, evidence-based psycho-educational booklet. It features comprehensive information on a range of topics identified from our previous research (McLoone et al. 2012) as important to people with melanoma. The booklet was developed by a multidisciplinary team with expertise in clinical psychology, psychological aspects of melanoma, dermatology, melanoma treatment and risk management, genetic epidemiology, public health, genetic counselling, patient education, and health economics. The booklet has seven modules and each module has been designed to stand alone, so that readers can use the different sections as needed. In addition, the booklet has integrated a series of resources tailored to people with melanoma such as:

- Graphics and diagrams to communicate risk information;
- Photographs to illustrate complex health behaviours such as skin self-examination;
- Verbatim quotes from Australian melanoma patients of various ages and backgrounds;
- A Question Prompt Sheet, which is a structured list of questions that patients are encouraged to ask their doctor if they wish;
- Care planning pages designed to provide patients with space to record various aspects of melanoma management, including: diagnosis, treatments, prognosis, skin biopsies, sentinel or lymph node biopsies, moles being monitored for change, and recommended follow-up care such as skin self-examinations and clinical skin examinations; and
- Up-to-date lists of reputable services and websites.

The booklet was reviewed and revised using an iterative process in partnership with two advisory panels involving consumers and health professionals. Representatives from key professional bodies such as the Australian Psychological Society, NSW Health, the Psycho-oncology Co-operative Research Group, and the Melanoma Institute Australia contributed to this process. The booklet was developed in accordance with the latest evidence, core principles of clinical psychology practice, and the Transactional Model of Stress and Coping (Lazarus & Folkman 1984; Folkman 1997). Development was also heavily guided by the NHMRC guidelines on ‘How to present the evidence for consumers’ (National Health and Medical Research Council (NHMRC) 1999), as well as relevant NHMRC clinical practice guidelines (i.e. the ‘Clinical practice guidelines for the management of melanoma in Australia and New Zealand’(Australian Cancer Network Guidelines Revision Working Party 2008), and the ‘Clinical practice guidelines for the psychosocial care of adults with cancer’) (Centre & Initiative 2003). In addition, the readability level of all materials was adjusted to 8^th^ grade, and pictorial representations of disease risk estimates were developed, as recommended by health communication experts (Elwyn et al. 2006).

In addition to the psycho-educational booklet, participants in the intervention group will receive *three individual, telephone-based sessions facilitated by a psychologist*. The three telephone sessions will occur at specific time points, timed according to participants’ HRC appointments:

- Session 1 will take place approximately one week before each participant’s next full dermatological appointment at the HRC.
- Session 2 will occur approximately one week after the HRC appointment.
- Session 3 will occur approximately three weeks after the HRC appointment (i.e. two weeks after Session 2).

As with the booklet, the telephone-based sessions have been developed in accordance with the latest evidence in melanoma and psycho-oncology research, (McLoone et al. 2013a) as well as core principles of brief psychodynamically-oriented psychotherapy (Abbass et al. 2009; Blagys & Hilsenroth 2002; Shedler 2010). The overarching framework of the intervention is to provide empathic, active listening at a deep level so as to try to understand the participant and his or her experiences, and to assist the participant in developing more effective emotional and behavioural coping strategies (De Jong & Berg 2002). The telephone intervention features seven key elements: 1. a focus on affect and the expression of patients’ emotions; 2. an exploration of patients’ attempts to avoid topics or engage in activities that may hinder understanding; 3. the identification of patterns in patients’ actions, thoughts, feelings, experiences, and relationships; 4. space to explore past experiences as well as future possibilities; 5. a focus on patients’ interpersonal experiences; 6. an emphasis on the therapeutic relationship; and 7. an exploration of patients’ needs, goals and wishes. Whilst this basic framework for the sessions will be outlined by the psychologist, the nature, scope and content of the sessions will be directed by the patient according to his or her unique and specific experiences, difficulties, needs, and wishes. The intervention is also designed to assist the participant in developing the skills and accessing the resources required to address identified difficulties, needs, and goals. While an explicit focus is not placed on fear of melanoma recurrence, based on previous research (Armes et al. 2009; Hodgkinson et al. 2007a; Sanson-Fisher et al. 2000) and our clinical experience it is expected that this will frequently arise as a difficulty experienced by patients. Hence, specific strategies in addressing FCR are included in the intervention (Butow et al. 2013), to be utilised as indicated. A comprehensive manual has been developed by the psychology team to provide detailed information and protocols relating to all clinical aspects of the trial.

The telephone sessions are designed to provide *patient-specific* assistance (see Table 1). Session 1 (up to 90 min) features an assessment, including a discussion of each participant’s background (family, work, friendships, …), experience of melanoma and clinical care, other health issues, information and support needs, and their goals and wishes for the intervention. Subsequent sessions (up to 50 min each) will focus on exploring each participant’s needs and concerns, utilising appropriate psychological techniques and the booklet, ‘Melanoma: Questions and Answers’. Session 2 will also include exploration of the participants’ experience of Session 1, his or her recent HRC appointment and its outcomes, the clinical care received, and related information and support needs. Session 3 will entail a summary of issues discussed during the previous two sessions, and the participant’s experience of working with a psychologist. Appropriate referrals will also be given for further information and support, as needed. The telephone sessions will be recorded with participants’ permission.

**Table 1:** Outline of the telephone sessions

	Content	Approximate duration
Introduction and scheduling of Session 1	• Therapist introduces herself to the participant over the telephone.	15 min
	• Check that both booklets have been received.	
	• Reiterate the aims of the intervention.	
	• Answer any questions the participant may have about the trial.	
	• Explain confidentiality.	
	• Schedule and set up Session 1.	
Session 1: Assessment	• Initial assessment of participants’ needs	90 min
	• Orient the participant to the different sections of the booklet, *Melanoma: Questions and Answers*, and the different ways in which he or she can use the booklet.	
	• Assist the participant in identifying his or her unmet information and support needs	
	• Discuss any concerns the participant may have about their upcoming HRC appointment.	
Session 2: HRC Appointment Follow-up	• Follow-up regarding HRC appointment.	50 min
	• Review of previous session and any difficulties discussed.	
	• Address participant’s unmet support and information needs, utilising the booklet where relevant.	
	• Provide information and referral for managing unmet needs, when appropriate.	
Session 3: Final session	• Follow-up and update since last session.	50 min
	• Review of previous session and any difficulties discussed.	
	• Discuss the degree to which unmet needs have been addressed.	
	• Discuss new strategies to address possible future concerns.	
	• Orient the participant to relevant services and resources in the booklet for possible future concerns.	
	• Facilitate referral for psychological intervention, if indicated.	

### Psychologists and intervention training

Psychologists recruited to the study to deliver the telephone-based sessions will be required to be registered psychologists, with at least five years clinical experience in a health-related setting, and to demonstrate a high level of empathic understanding and communication in a role-play evaluation with a simulated patient. Once recruited to the trial (and prior to intervention delivery), the psychologists will undertake a tailored training program featuring five core learning components:

1. *Education in issues relating to melanoma and melanoma risk management.*
  Psychologists will be trained in physical, emotional, social, behavioural, cognitive and practical issues commonly experienced by people affected by melanoma by a senior member of the research team who is a psychologist with many years of experience in the care of people with melanoma (NK). Psychologists will also be supported in delivering the intervention through intensive training with the manual by an experienced psycho-oncology researcher and psychologist.
2. *Observation of routine clinical practice at the HRCs.*
  To facilitate an in-depth understanding of the nature and function of the HRCs and the clinical interactions that occur between patients and their healthcare team, psychologists will undertake a series of observations at different times and at different HRCs.
3. *Training in the delivery of telephone-based psycho-educational interventions.*
  The psychologists will undergo a training workshop designed to facilitate communication skills in the delivery of telephone-based psychological interventions for cancer patients.(Hodgkinson 2008; Shaw et al. 2013) The psychologists will also undertake a role-play with a professional actor from the Pam McClean Cancer Centre (www. http://pammcleancentre.org/) to help them practice and strengthen their telephone-based skills. The scenario for this role play has been written by two senior psychologists in the team.
4. *Clinical workshops.*
  A number of clinical workshops will be made available to the psychologists throughout the trial, to support their ongoing professional development.
5. *Weekly supervision.*
  Throughout the entire intervention, psychologists will attend weekly clinical supervision with a senior psychologist (NK).

### Study procedures

**Fig. 2:**
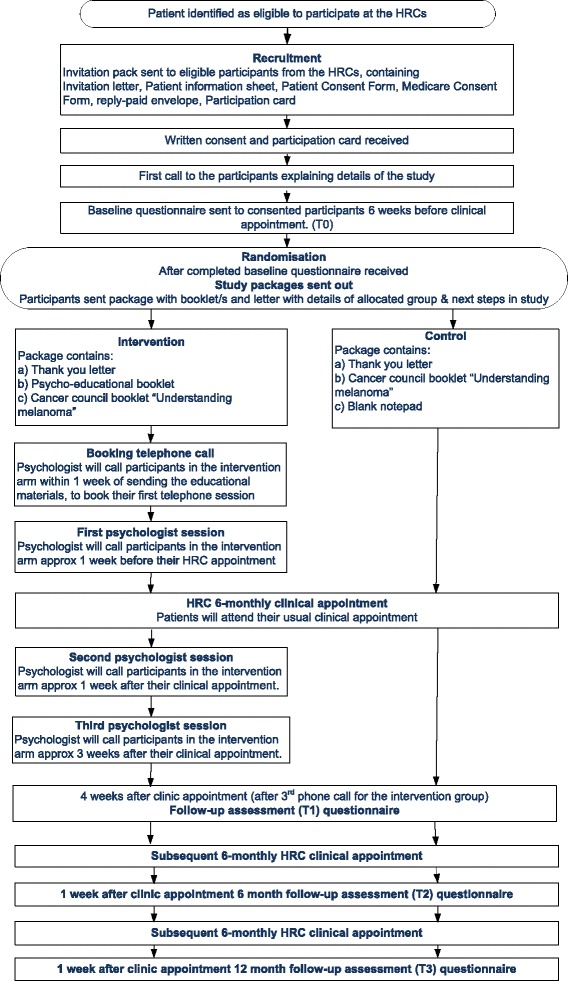
Flow diagram

Figure 2 illustrates the procedures of the trial. Once consented, participants will be asked to complete a paper- or web-based baseline questionnaire, depending on their preference, approximately 6 weeks before their next 6-monthly dermatological consultation at the HRC. Once the completed baseline questionnaire is received, the participant will be randomised into either the intervention or control group. After randomisation, 3–4 weeks before their HRC appointment, the research team will send the participant the appropriate study package, including a letter describing which group they have been randomly allocated to, and the next steps in the study. In the following week, participants in the intervention arm will be contacted by telephone by the assigned psychologist to arrange a suitable time for the first intervention session, and all three sessions will be facilitated by the same psychologist.

All participants will receive four study questionnaires in total; one at baseline (T0, about 6 weeks prior to their next full dermatological consultation at the HRC), one 4 weeks after their HRC consultation (which occurs soon after their third telephone session for those in the intervention group) (T1), and again 1 week after their subsequent full HRC consultation 6 months later (T2), and 12 months later (T3) (see Fig. 2). The 12 month assessment (T3) will assess longer-term effects of the intervention, but is only planned if effectiveness of the intervention is demonstrated at 6 months. Participants who do not complete and return the study questionnaires in the specified time period will be contacted by the research team via telephone or email, as a reminder about the study.

### Outcome measures

The primary outcome will be based on the third questionnaire at 6 months (T2).

### Effect evaluation

Table 2 provides an overview of the measures of efficacy, economic evaluation, and timing of measurement.

**Table 2:** Measures and timing of assessment in the Melanoma care study

Variables	Measures	Baseline (T0)	T1^*^	T2^Φ^	T3^‡^
Fear of new or recurrent melanoma	Fear of Cancer Recurrence Inventory	✓	✓	✓	✓
Depression, anxiety, stress and depression	Depression Anxiety and Stress Scales (DASS-21)	✓	✓	✓	✓
Health related quality of life	AQOL-8D and FACT-M	✓		✓	✓
Knowledge	A 9 item scale specifically developed for this study	✓	✓	✓	✓
Healthy behavioural adjustment to melanoma risk	The Sun Protection Habits Scale (SPHS) and adapted items for sun protection and skin self examination	✓		✓	✓
Satisfaction with clinical care	Consultation Satisfaction Questionnaire (CSQ)	✓	✓	✓	
Unmet information and support needs	Modified Cancer Survivors’ Unmet Needs (CaSUN)	✓	✓	✓	
Resource use and cost outcomes	Items developed for this study (resource use) and Medicare data (PBS and MBS)	✓		✓	✓
Health literacy		✓			
Medical record	From the melanoma clinics’ databases	✓		✓	✓
Process evaluation	Developed for use in this study.	✓		✓	
Demographic and other risk factors	Age, gender, marital status, number of children, education, income, occupation	✓			

^*^T1: 6 weeks after randomisation, ^Φ^T2: 7 months after randomisation, ^‡^T3: 13 months after randomisation

#### Primary outcome measure

The primary outcome in this study is the level of self-reported fear of new or recurrent melanoma assessed using the severity sub-scale of a modified (i.e. melanoma-specific) version the 42-item Fear of Cancer Recurrence Inventory (FCRI). (Simard & Savard 2009) A higher FCRI score is indicative of greater FCR. The other six sub-scales of the FCRI (triggers, psychological distress, functional impairment, reassurance, insight, and coping strategies) will also be analysed as outcomes. Previous research has demonstrated high internal consistency (α = 0.75–0.91) and reasonable temporal stability (r = 0.58–0.83) of the sub-scales of the FCRI, as well as good criterion validity when compared with other self-report scales assessing fear of cancer recurrence (r = 0.68 to 0.77) or related constructs (Simard & Savard 2009).

#### Secondary outcome measures

*General depression, anxiety and stress*: measured using the short version of the Depression Anxiety and Stress Scales (DASS-21) (Lovibond & Lovibond 1995). The DASS-21 is a set of three 7-item self-report scales designed to measure the emotional states of depression, anxiety and stress. The total score of each subscale of the DASS-21 is classified from “normal” to “extremely severe”.

*Health-related quality of life (HRQoL)*: HRQoL will be assessed using two measures. The Assessment of Quality of Life—8 Dimensions (AQoL-8D) is a 35-item health-related QoL preference-based measure, specifically developed for mental health, with norms for the Australian population by age and sex. The AQOL-8D produces utilities for quality-adjusted life year (QALY) estimation, used in economic evaluations (Richardson et al. 2009; Richardson & Iezzi 2010). The Functional Assessment of Cancer Therapy (FACT-M) (Cormier et al. 2005) (melanoma) contains the 27 core items of the FACT–G (general), plus an additional 24 melanoma-specific items encompassing three domains: physical well-being (20 items), emotional well-being (3 items), and social well-being (1 item). The FACT-M yields both a total QoL score as well as domain scores. An algorithm is available to transform FACT-M scores to utilities for QALY estimation, thereby facilitating a comparison of health economic outcomes.

*Behavioural adjustment to melanoma risk*: Three melanoma-related behaviours will be assessed: sun exposure, sun protection, and skin self-examination. Three items from the consensus-based set of core survey items developed by Glanz et al. will be used to assess sun exposure (Glanz et al. 2008). One item will assess instances of sunburn in the past 12 months. The Sun Protection Habits Scale (Glanz et al. 2002) will be adapted to assess the use of seven sun protection behaviours (e.g. sunscreen use). Engagement in skin self-examination will be assessed with three items adapted from Manne et al. (Manne & Lessin 2006).

*Satisfaction with clinical care*: will be measured using the Consultation Satisfaction Questionnaire (CSQ) (Baker 1990). The CSQ is comprised of 18 items assessing: general satisfaction (3 items); professional care (7 items); depth of relationship (5 items); and perceived time (3 items).

*Unmet information and support needs*: will be assessed using an adapted version of the Cancer Survivors’ Unmet Needs (CaSUN) questionnaire, which includes needs related to information, medical, emotional, quality of life, life perspective (Hodgkinson et al. 2007b; Hodgkinson et al. 2007c). The CaSUN includes 35 unmet need items, 6 positive change items and an open-ended question.

*Knowledge*: A purposely-designed set of items was developed to assess knowledge of specific issues covered in the booklet ‘*Melanoma: Questions and Answers’.*

#### Covariates

Information on demographic factors including age, sex, marital status, education, number of children, health literacy and total family income will be collected. In addition, clinical information such as their doctor’s name, presence of dysplastic nevus syndrome, presence of high penetrance genetic mutation, family history of melanoma, dates of personal melanoma diagnoses, melanoma American Joint Committee on Cancer (AJCC) stage, Breslow thickness, site of the melanoma(s), and other major illnesses will be extracted from the clinic records.

### Sample size

Sample size calculation has been based on 80 % power and a two-sided α = 0.05 test. Because there is no existing literature or clinical opinion to provide an estimate of the minimal clinically important difference, a standardised mean difference (Cohen’s *d*) of 0.5 was employed, which is a moderate effect size and has been found to be applicable to a wide variety of patient-reported outcomes (Norman et al. 2003). Based on these values, the sample size required is 64 per group. When taking into account the expected attrition rate of 20 %, the sample size required is 77 per group (154 total).

### Economic evaluation

A trial-based economic evaluation will be conducted from a health system perspective. The following items for measurement of costs will be identified, measured and valued:

- Cost of the production of the psycho-educational booklet;
- Psychologist costs;
- Number of telephone sessions and duration;
- Access to external psychological services;
- Hospital admissions; and
- Prescribed medications.

Health service use and costs of the intervention will be estimated using three methods: 1) the Australian Medicare Benefits Schedule (MBS) and Pharmaceutical Benefits Scheme (PBS) databases (individual informed consent was obtained to access to these individual-level data); 2) self-report regarding use of healthcare resources not covered by the MBS and PBS databases; and 3) trial records, including research team and psychologist records related to intervention delivery.

In the economic evaluation, total and mean costs for the intervention and control group will be reported in a disaggregated format. Total and mean severity outcomes (FCRI Severity subscale score and AQOL-8D utility (quality of life score)) will be reported for the intervention and control groups. The difference in mean scores between the two groups will be assessed with appropriate statistical tests. Incremental cost-effectiveness ratios will be calculated in terms of: a) the incremental cost per participant achieving a significant decrease in mean FCRI Severity score (0.5 of the SD on the FCRI Severity subscale which is considered to be a meaningful change) (Norman et al. 2003); and b) the incremental cost per quality-adjusted life year (QALY) gained in the intervention group compared with the control group. Results will be plotted on a cost-effectiveness plane. Bootstrapping will be used to estimate a distribution around costs and health outcomes and to estimate the confidence intervals around the incremental cost-effectiveness ratio (Medical Services Advisory Committee MSAC 2000; Drummond et al. 2005; Gold et al. 1996). One-way sensitivity analysis will be conducted around key variables including the QoL instruments, and a cost-effectiveness acceptability curve (CEAC) will be plotted (Medical Services Advisory Committee MSAC 2000; Drummond et al. 2005; Gold et al. 1996).

### Process evaluation

A process evaluation will also be undertaken to: a) assess intervention fidelity and reach; b) assess participants’ satisfaction with and acceptability of the intervention; and c) provide data to assist in interpreting the outcomes of the trial. These process evaluation questions have been developed by operationalising key elements of process evaluations (as described by Baranowski and Stables, and Linnan and Steckler (Baranowski & Stables 2000; Linnan 2002; Saunders et al. 2005)), into structured items. Data will be collected using questionnaires completed by participants, listening to a selection of the recorded telephone sessions checklists and notes kept throughout the trial by psychologists, and research notes. Descriptive statistics, Chi-square and t-tests will be used to analyse data from the process evaluation.

### Statistical analyses

The primary analysis will be by intention to treat. For both primary and secondary endpoints, linear mixed models will be used to analyse the data to account for the repeated measures (and therefore non-independent) data collected from patients. These models will enable the comparison of potential changes in patient responses over time, whether the treatment and control arms differ from each other, and whether any changes over time differ between the treatment arms (that is, whether there is a time-by-treatment interaction). Several socio-demographic variables (e.g. age, sex, income, education level, marital status) will be statistically controlled for by appropriate inclusion as covariates in the mixed models.

## Discussion

### Significance

To our knowledge, the Melanoma Care Study will be the first randomised clinical trial to test an intervention specifically aimed at improving the psychological health and well-being of melanoma survivors at high risk of developing new primary disease. The study will investigate how the newly-developed intervention influences psychosocial outcomes reported by people affected by melanoma, with a focus on FCR, psychological health and well-being, melanoma-related actions and behaviours, unmet information and support needs, satisfaction with clinical care, risk-related knowledge and understanding, and cost-effectiveness. This trial aims to strengthen the literature and to bridge the gap between existing research evidence demonstrating a critical need for psycho-educational support for melanoma patients, and clinical practice.

### Strengths

The Melanoma Care Study has several strengths.

First, the newly developed intervention is based on extensive previous research by our group and others (McLoone et al. 2013a; McLoone et al. 2012; McLoone et al. 2013b) identifying a range of psychological needs and experiences reported by high risk melanoma survivors. These experiences include: high levels of fear regarding melanoma recurrence or the development of new primaries, low confidence and skill in skin self-examination, limited access to appropriate and timely psychological care, limited use of effective coping strategies, difficulties understanding and interpreting complex medical information, and challenges associated with satisfaction with clinical care. A wide range of evidence-based features have been integrated into the intervention to address these common difficulties. In addition, the timing of the support sessions has been based around patients’ full dermatological appointments at the HRC, as this is a time of peak anxiety for patients. For example, a recent study by Morton et al. on melanoma patients’ experiences of follow-up found that anxiety was a major concern starting from one week prior to clinical visit (Morton et al. 2013). Third, the systematic recruitment of large numbers of high risk individuals through the HRCs provides a unique opportunity to address the research questions with sufficient power and in a timely manner. Fourth, the investigation of cost-effectiveness provides evidence to support policy-makers regarding adoption and reimbursement of the intervention, as previous research has found economic evaluations are often overlooked in intervention research (Badr & Krebs 2013). Fifth, the conduct of a process evaluation alongside the trial adds value and can facilitate translation and implementation of study results, if positive, into future clinical practice. Research has demonstrated that process evaluation can play an important role in the evaluation of complex interventions (Campbell et al. 2000) and the process data can be useful when trying to explain why an intervention may work or fail to work (Elford et al. 2001; Stapleton et al. 2002).

### Limitations

Despite these strengths, the study presents several challenges. The first challenge is whether the scale used to measure the primary outcome (FCRI) will have the sensitivity required to detect any changes experienced by this particular cohort. To overcome this challenge, the FCRI has been subjected to confirmatory factor analysis and item response theory analysis in order to examine its measurement properties in melanoma survivors (submitted for publication). Another challenge of the study is that participants were not screened for any psychological variable (e.g. FCR, anxiety or depression) before trial enrolment. Indeed, patients with no information or support needs may perceive a lack of usefulness of the intervention which may influence the number of participants who withdraw from the study. However, the intervention was broadly designed to meet the supportive care needs of melanoma survivors at high risk, and FCR was one part of the overall suite of issues that we wanted to address.

Furthermore, the psychologists have been trained to work with participants to identify needs and tailor the intervention accordingly.

### Future implementation

In 2008, the NHMRC Clinical Practice Guidelines for the Management of Melanoma recommended that psycho-education be made available to all patients with melanoma (Australian Cancer Network Guidelines Revision Working Party 2008). This signals a high potential for rapid translation of effective interventions into the clinic, if evidence for the efficacy of this intervention in improving psychological health and well-being is found, and it is also cost-effective.

## Abbreviations

AJCC: American Joint Committee on Cancer
AQoL-8D: Assessment of Quality of Life – 8 Dimensions
CaSUN: Cancer Survivors’ Unmet Needs
CEAC: cost-effectiveness acceptability curve
CSQ: Consultation Satisfaction Questionnaire
DALYs: Disability-Adjusted Life Years
DASS: Depression Anxiety and Stress Scales
FCR: Fear of Cancer Recurrence
FCRI: Fear of Cancer Recurrence Inventory
FACT-M: Functional Assessment of Cancer Therapy
HRQOL: Health Related Quality of Life
HRC: High Risk Clinic
MBS: Medicare Benefits Schedule
NHMRC: National Health and Medical Research Council
PBS: Pharmaceutical Benefits Scheme
QALY: Quality-Adjusted Life Year
SPHS: Sun Protection Habits Scale
